# Solution combustion synthesis of ZnO doped CuO nanocomposite for photocatalytic and sensor applications

**DOI:** 10.1038/s41598-024-82764-2

**Published:** 2025-01-02

**Authors:** A. Naveen Kumar, M. Balakrishna, Usha Desai, R. Rakshith, K. M. Ambika, P. Soumya, C. R. Ravikumar, S. Senthil Vadivu, Nithesh Naik

**Affiliations:** 1 Department of Physics, S.E.A College of Engineering and Technology, Bengaluru, Karnataka 560049 India; 2 Department of Mathematics, S.E.A College of Engineering and Technology, Bengaluru, Karnataka 560049 India; 3 Department of Electronics and Communication Engineering, S.E.A College of Engineering and Technology, Bengaluru, Karnataka 560049 India; 4 Department of Chemistry, S.E.A College of Engineering and Technology, Bengaluru, Karnataka 560049 India; 5 Research Centre, Department of Chemistry, East West Institute of Technology, Bengaluru, Karnataka 560091 India; 6 Department of Computer Science Engineering (IoT-CSBT), S.E.A College of Engineering & Technology, Bengaluru, Karnataka 560049 India; 7 Department of Mechanical and Industrial Engineering, Manipal Institute of Technology, Manipal Academy of Higher Education, Manipal, Karnataka 576104 India

**Keywords:** Solution combustion, ZnO-CuO nanocomposites, Photocatalytic activity, Cyclic voltammetry, Sensors, Materials science, Nanoscience and technology, Physics

## Abstract

ZnO-doped CuO nanocomposites (CuO-ZnO NPs) of 1, 3, and 5 mol% were prepared by the solution combustion method using ODH as a fuel (Oxlyl-hydrazide) at 500 °C and calcining at 1000 °C for two hours and the Structural, photocatalytic, and electrochemical properties were investigated by experimental and theoretical methods. X-ray diffraction (XRD) patterns revealed a crystallite size (D) range of 25 to 31 nm for pure CuO and 1, 3, and 5 mol% CuO-ZnO NPs. According to calculations, the optical energy band gap (Eg) of the NPs is between 2.1 and 2.5 eV. Under UV light irradiation, the photocatalytic degradation of CuO + 3%ZnO NPs on Congo Red (CR) and Methylene Blue (MB) dye was assessed under the influence of UV light. The degradation efficiency increased with the catalyst dosage (10 − 60 mg L^− 1^). At a concentration of the catalyst of 60 mg, the degradation efficiency can even reached 70% after 120 min. The electrochemical properties of the prepared NPs were studied using cyclic voltammetry and electrochemical impedance spectroscopy (EIS). Solutions of glucose and ascorbic acid were effectively sensed using modified carbon paste electrodes. These innovative results can be considered for the expansion of novel resources to scale for dual applications in the areas of photocatalysis and sensors.

## Introduction

To fulfil the world’s rapidly increasing energy demands, it is imperative that the development of high-performance, affordable, environmentally acceptable renewable energy storage and conversion technologies is accelerated^1–3^. Ultracapacitors and supercapacitors are two types of electrochemical capacitors that are used in energy storage devices. Owing to their unique characteristics, which include better power density and longer cyclic life than traditional batteries, higher energy density capacitors, high rate capacity, quick charge and discharge mechanisms, and eco-friendliness, they have drawn more attention in recent years^4–8^. Supercapacitors are arguably the most significant emerging energy-storage technology^9^. Supercapacitors have drawn increasing attention because of their many applications in a variety of industries, including factory power backup, electric vehicles, load cranes, forklifts, and electric utilities^10^. Supercapacitors are divided into two categories based on their charge storage methods and types of electrode materials: electric double-layer capacitors (EDLCs) and pseudo-capacitors. In EDLCs, charges can be electrostatically stored owing to ion adsorption at the electrode-electrolyte interface. Carbon-based electrode materials include graphene, carbon aerogels, carbon nanotubes, and activated carbon (AC)^11,12^. To store charges resulting from electrochemical redox reactions, pseudocapacitors are utilized with transition metal oxides (CuO, CuO, RuO_2_, MnO, NiO, and CoO)^13–15^ or conducting polymer electrode materials (polypyrrole, PANI)^16,17^. The benefits of EDLCs and pseudocapacitors, particularly their high energy and power densities, are combined in hybrid supercapacitors. Most interest has been drawn to the unique magnetic, catalytic, and electrical properties of metal-oxide nanoparticles^18^. RuO_2_ has emerged as one of the most promising choices among the transition-metal oxides. However, the high cost and toxicity of RuO_2_ limit its use^19^. Therefore, it is essential to develop sustainable alternatives. Excellent redox characteristics make the metal oxide copper oxide (CuO) useful for many different applications. CuO has been studied because of its unique features, including low toxicity, good electrochemical properties, abundance in nature, low cost, outstanding catalytic activity, ease of synthesis, eco-friendliness, and variety of nanoscale morphologies^20–22^. CuO has been employed in electrochemical processes as an electrode material in solar systems, heterogeneous catalysts, lithium-ion batteries, supercapacitors, and gas sensors^23^. It has been selected as one of the components of nanocomposite electrode systems because of its improved electrochemical properties. In the present study, we doped CuO with different concentrations of ZnO to improve the catalytic properties of CuO nanoparticles. CuO and ZnO generally have better chemical, mechanical, and physical properties, lower melting temperature, high diffusion, structural stability, larger surface area, and high surface energy^24^. An n-type semiconductor with a 3.37 eV broad band gap energy, such as ZnO, and a p-type semiconductor with a 2.5 eV narrow band gap energy, such as CuO, occur when linked^25,26^. The need for high-purity CuO for industrial applications has led to the development of a wide range of synthesis techniques, from basic thermal oxidation, electrochemical, precipitation, and hydrolysis to extremely complex methods such as sol-gel, microemulsion, solvo-thermal, and microwave hydrothermal methods. To influence the different elements of the synthesized nanomaterial, these strategies aim to modify the composition, particle size, crystalline structure, shape, and functional stability^27^. By adjusting the concentration of the starting materials and doping agents used in a specific synthesis procedure, the properties of nanomaterials can be modified. In the present work, we have detailed the sonochemical synthesis of CuO nanoparticles in the present work. This preparation procedure has attracted attention because of its homogenous mixing, reaction control conditions, and management of particle size distribution during ultrasonic exposure^28^. CuO NPs were doped with ZnO at different concentrations to generate composite materials for photocatalytic and supercapacitor applications. The modified CuO-ZnO NPs electrode was tested as a supercapacitor electrode, and the rate at which direct green and rapid blue degraded in the presence of UV light was used to gauge the photocatalytic activity of each composite^29^. These novel findings, in our opinion, are highly important because they could facilitate the effective synthesis of these and related metal oxide semiconductors and provide crucial data that will further our understanding of their photocatalytic activity. In many different applications, such as the cosmetic, textile, ink, and leather industries, dyes are the most widely utilized organic compounds; globally, they account for approximately 60–70% of dye production. Reports state that CuO. Drinking water, aquatic life, and marine areas are all seriously threatened by the introduction of dyes into water supply. A few noteworthy examples in this regard are the carcinogenicity and mutagenicity of azo dyes and their derivatives^30^. Consequently, dye removal from effluents has been the subject of in-depth research for many years. Despite the near-impossibility of completely eliminating organic dyes and residual colors, many efforts have been made to clean up effluents that have been contaminated with these dangerous substances. Numerous physical and chemical methods have been studied, including adsorption and photocatalysis. Because of its ease of use, reasonable efficiency, and neutrality towards the chemicals present in wastewater, adsorption has been widely regarded as a highly effective method for reducing the degree of pollution^31^. The use of this method for dye removal dates back to the early 1900s, when it was necessary to eliminate each dye molecule separately to reduce the risk to water supply. However, in recent years, creating treatments that are both reasonably priced and effective has become crucial. The application of natural materials and biosorbents made from agricultural waste has attracted attention from this perspective. Sensors are essential for real-time monitoring of various parameters within energy storage systems, such as temperature, voltage, current, and state of charge (SoC). By providing accurate and timely data, sensors enable better management of energy flows, ensuring optimal performance and longevity of storage systems. For instance, temperature sensors help prevent thermal runaway in lithium-ion batteries by monitoring and managing the heat generated during charging and discharging cycles. Voltage and current sensors provide critical information for balancing the load and detecting faults, thereby enhancing the safety and efficiency of the system^31,32^.

## Materials and experimental methods

The Stoichiometric ratios of analytical grade chemical precursors, such as Cu(NO_3_)_2_.3H_2_O, (Zn(NO_3_)_2_.6H_2_O) with 99.99% of purity purchased from Sigma Aldrich and used without further purification. The fuel ODH prepared from the lab was used directly for heterostructure nanocomposite synthesis.

###  Synthesis of CuO–ZnO hybrid nanocomposite

**Fig. 1:**
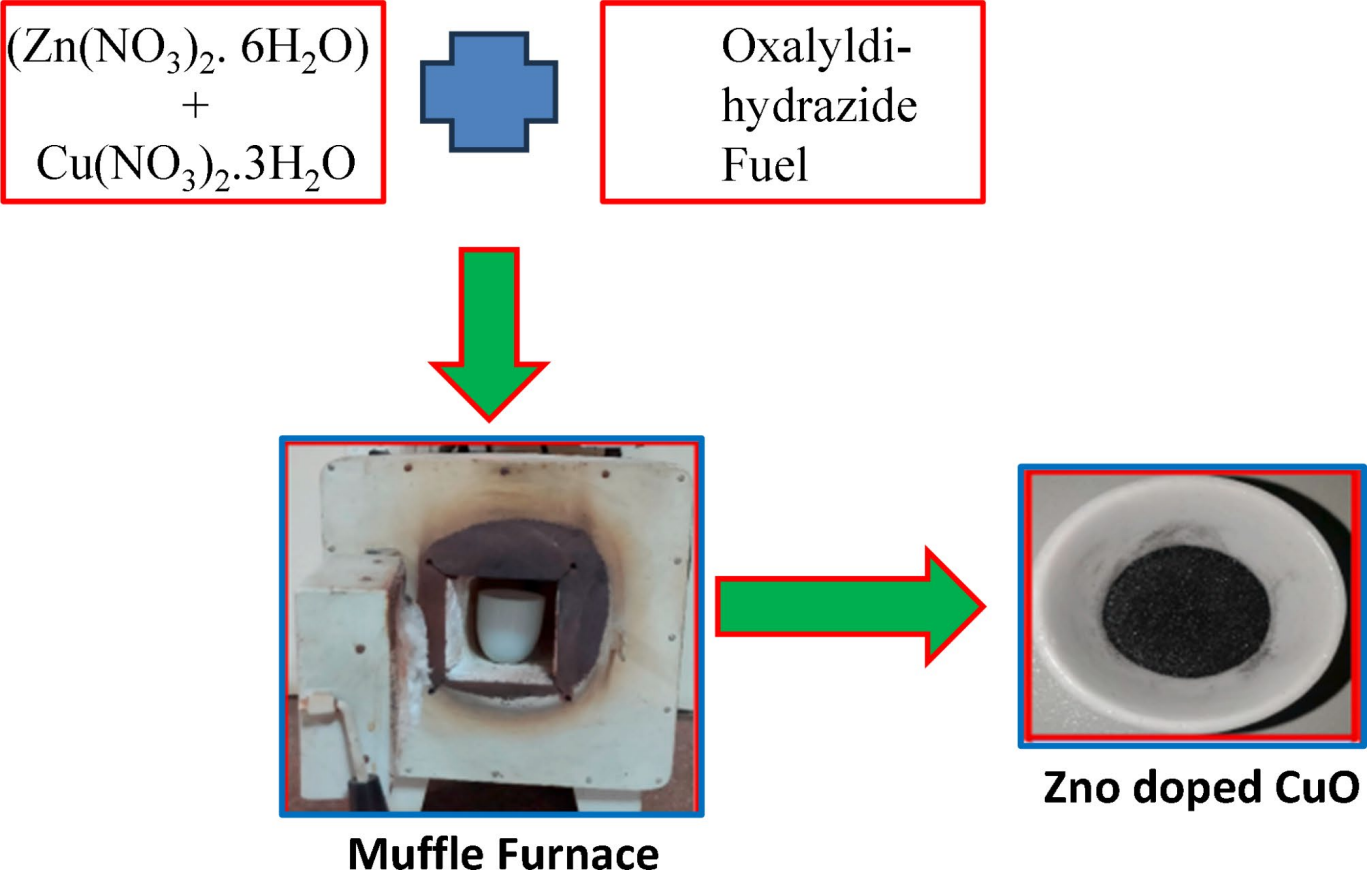
Schematic experimental representation of synthesis of ZnO doped CuO nanocomposite by solution combustion route.

The stoichiometric ratios of the analytical-grade chemical precursors Cu(NO_3_)_2_.3H_2_O, (Zn(NO_3_)_2_. 6H_2_O) and ODH prepared in the lab (ratio of 1.85 (F/O)) were used to synthesize the CuO–ZnO nanocomposite with no further purification. The starting material was mixed with a minimal quantity of water in a silica crucible and continuously stirred to attain homogeneity. Further, the above solution mixture was placed in a muffle furnace preheated to 500 ± 5◦C and calcined at 1000^0^c for 2 h. The foam as-formed ZnO-doped CuO nanocomposite formed within 5 min was cooled down to room temperature, dried, and collected. The solution combustion method was followed by the aforementioned procedure to synthesize 1,3,5 mol% ZnO-doped CuO nanocomposites^33^. A schematic representation of the synthesis of the ZnO-doped CuO nanocomposites is shown in Fig. 1.

###  Photocatalytic measurement

The photocatalytic study was carried out at room temperature in a circular glass container with 169.8 cm^2^ surface area. In a wide glass bowl, 40 mg of the synthesized nanoparticles was mixed with 20 ppm CR and MB dye solution. Photocatalytic experiments were carried out separately for both dyes for approximately 60 min under natural sunlight irradiation. At regular time intervals, 5 ml of the study solution was removed, and the absorption rate was monitored. All of the experiments were carried out in triplicate, with errors of less than 3%, and the average value was recorded.

###  Preparation of working electrodes

For the preparation of carbon paste electrode, 500 mg of silver decorated graphite powder was thoroughly mixed with 20% of silicone oil. The resulting paste was packed into a Teflon tube, and a copper wire was inserted for external electrical contact. The surface was polished using butter paper. When necessary, a fresh surface was obtained by mechanically pushing the excess and polishing the electrode surface using a steel rod. CH Instrument (CHI-608E) in a 1 M KOH electrolyte.

##  Results and discussions

###  PXRD analysis

**Fig. 2:**
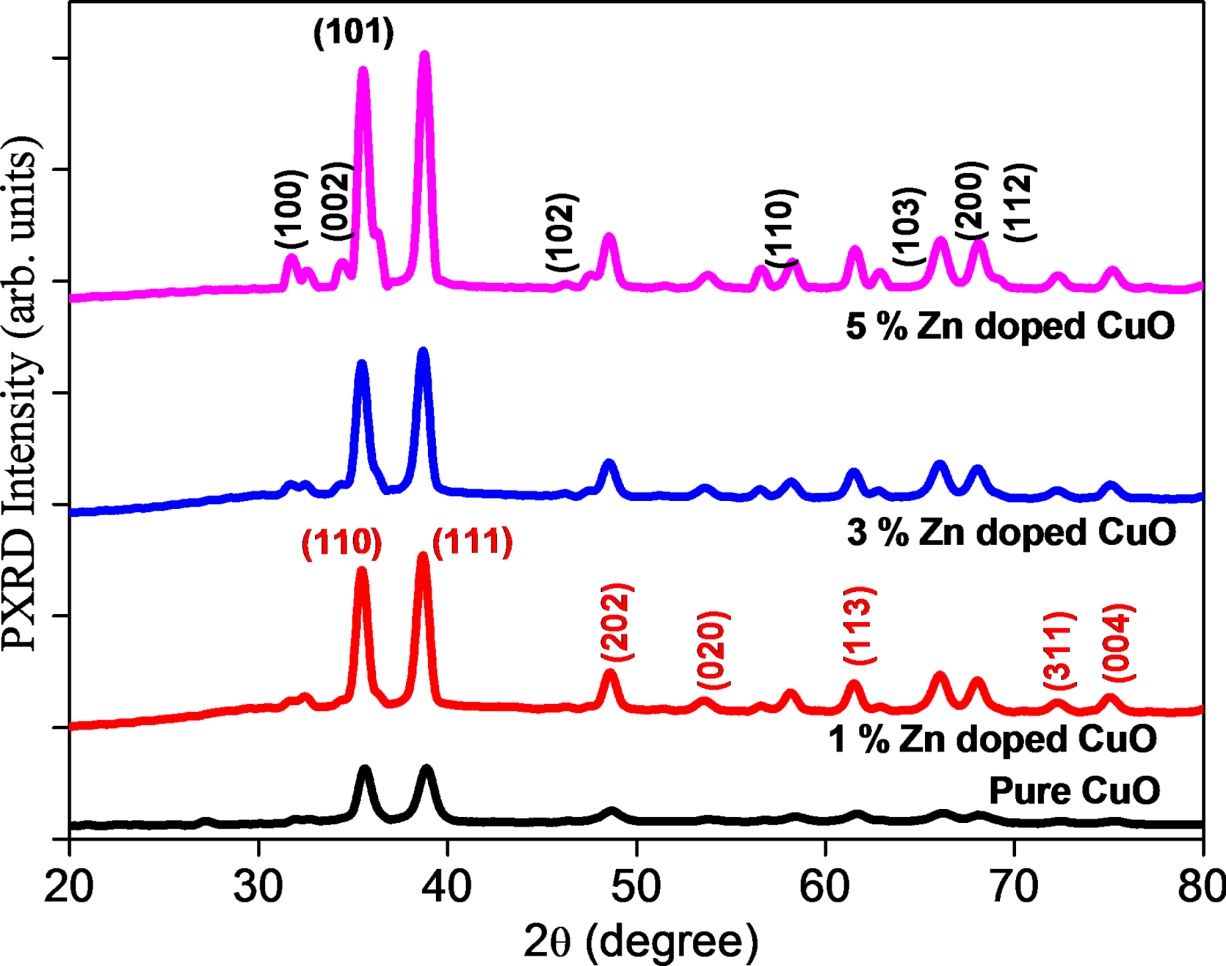
PXRD spectra of ZnO-CuO(1,3,5%) Nanocomposites.

The PXRD patterns of combustion-synthesized ZnO-doped CuO nanoparticles are shown in Fig. 2. The diffraction pattern obtained confirms the formation of the ZnO-doped CuO phase. diffraction peaks were indexed with standard reference pattern JCPDS 36-1451 for pure and JCPDS Card No: 00-001-1136 for CuO + 1% ZnO with a nanorod structure^34^. The 2θ values of the diffraction peaks correspond to the (100), (002), (101), (111), (102), (110), (103), (200), and (122) planes, respectively. The narrow and high-intensity peaks confirmed the high crystallinity and small dimensions of the synthesized nanoparticles. From the Debye-Scherrer equation, the average size of crystalline ZnO-doped CuO nanoparticles was calculated to be 23 nm, as shown in Table 1.

1 $$D=\frac{K\lambda}{\beta cos\theta}$$

Wherein, ‘K’- constant, ‘λ’-wavelength and ‘β’-full width at half maximum (FWHM).

**Table 1:** PXRD spectra of ZnO doped CuO Nanocomposites.

2θ	θ	FWHM	Crystallite size	Plane (hkl)
35.47	17.735	0.225	23.81	(110)
38.72	19.36	0.442	22.7	(111)
48.65	24.325	0.375	22.15	(202)
53.65	26.825	0.423	22.78	(020)
61.58	30.79	0.248	22.71	(113)
72.13	36.065	0.356	23.2	(311)
75.12	37.56	0.421	23.1	(004)

### Scanning electron microscopy studies

**Fig. 3:**
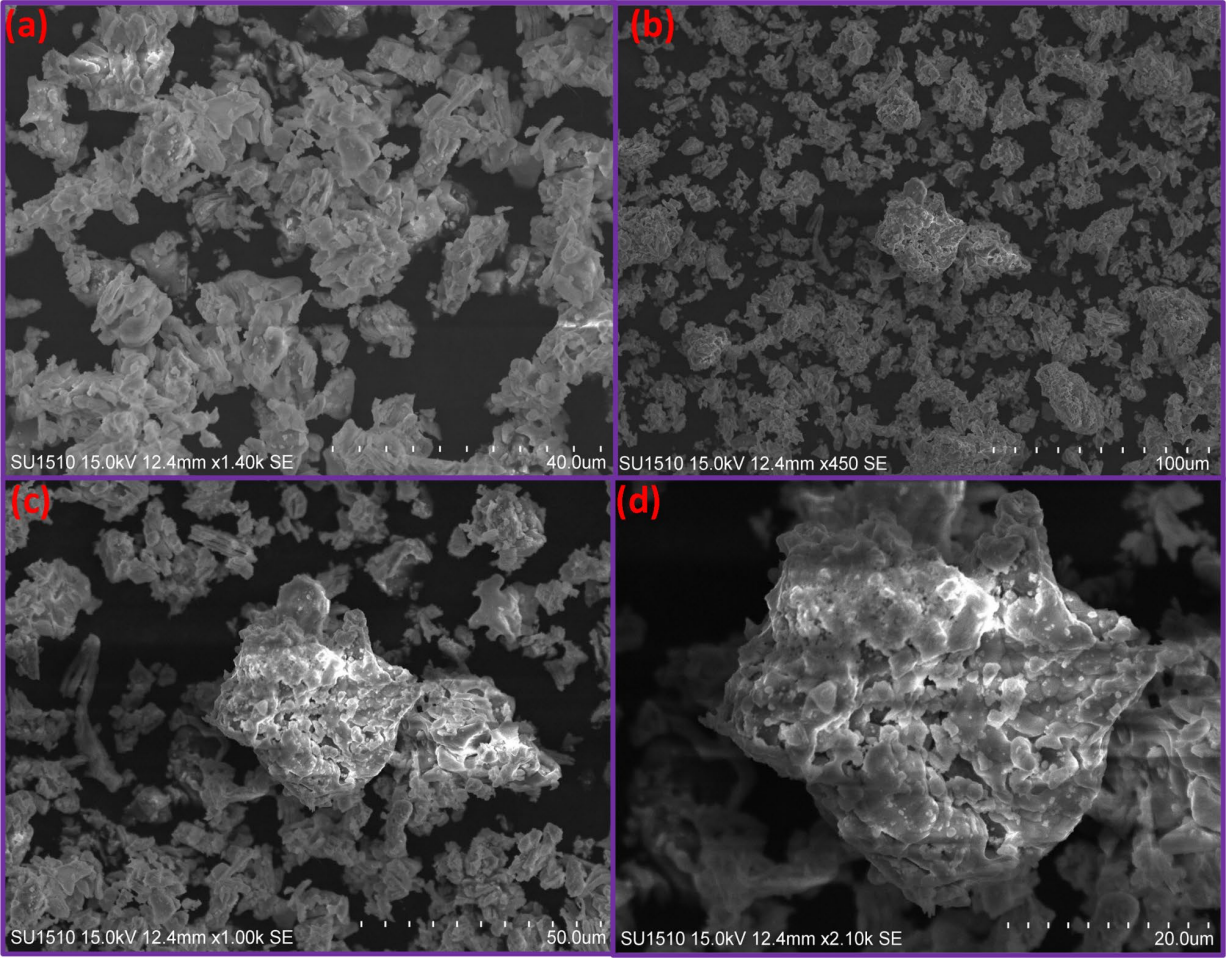
SEM micrographs of (**a**) CuO, (**b**) CuO + 1% ZnO, (**c**) CuO + 3%ZnO and (**d**) CuO + 5%ZnO.

The SEM images in Fig. 3(a–d) show the morphology of the synthesized 1, 3, and 5 mol% ZnO-doped CuO nanocomposite (CuO-ZnO NPs) with different compositions. The prepared samples showed irregular shapes because of the uneven distribution of temperature and mass flow in the combustion flame. Furthermore, the more porous nature was attributed to the escape of gases with high pressure during combustion^35^.

###  Diffusion Reflectance spectroscopy (DRS) studies

**Fig. 4:**
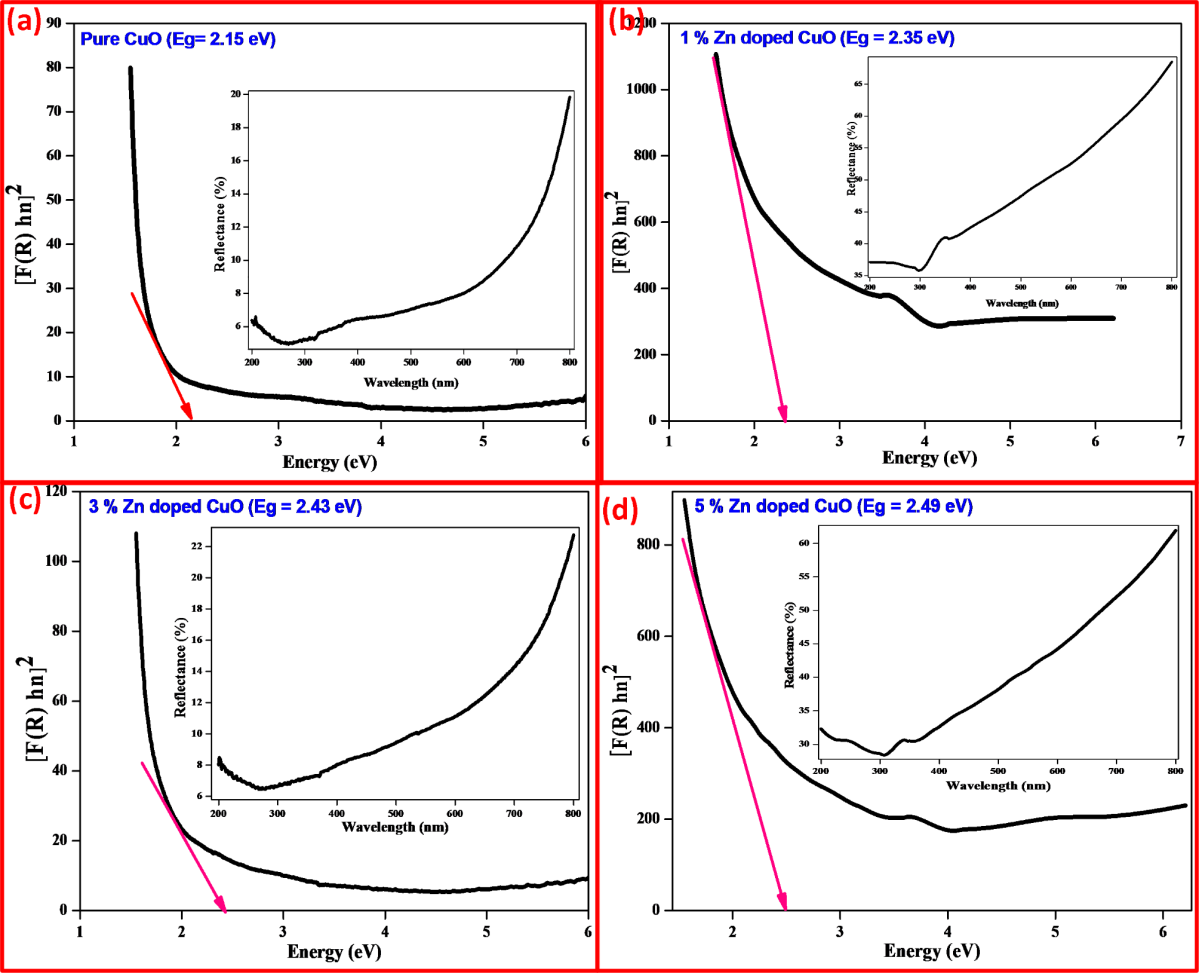
**(a-d)** DRS and Energy band-gap spectra of samples (**a**) CuO, (**b**) CuO + 1% ZnO, (**c**) CuO + 3%ZnO and (**d**) CuO + 5%ZnO.

The conducting characteristics of synthetic ZnO-CuO(1,3,5%) nanocomposites with different molar ratios in the 200–800 nm range were evaluated using DRS analysis. The DRS analyses of the ZnO-CuO(1,3,5%) photocatalysts modified using the solution combustion approach are shown in Fig. 3. The Kubelka-Munk method was used to quantify the reflecting band gap of the synthesized ZnO-CuO(1,3,5%) photocatalysts in Fig. 4(a-d), as well as the connection between the scattering coefficient (S), absorption coefficient (K), and diffuse reflectance of the ZnO-CuO(1,3,5%) NPs^36^.

2 $$\:F\left(R\right)=\frac{{(1-R)}^{n}}{2R}=\frac{K}{S}$$

n determines whether a transition is allowed directly (n = ½) or indirectly (*n* = 2)^37,38^. The linear absorption coefficient (α) and energy band gap of a material can be obtained using the Tauc chemical relation, which is given by:

3 $$⍺=\frac{{{C}_{1}(h\nu-Eg)}^{\frac{1}{2}}}{h\nu}$$

4 $${\left[F\left(R\right)h\nu\:\right]}^{2}={C}_{2}\left[h\nu\:-Eg\right]$$

From the [F(R)hv]^2^versus hν plot, the linearly fitted regions were also projected as [F(R)hv]^2^=0 to determine the value of Eg. The band gap energy of the synthesised ZnO-CuO(1,3,5%) nanocomposites was found to be 2.16,2.36,2.48 & 2.48 eV, respectively (Fig. 4a–d).

###  Photocatalytic studies

**Fig. 5:**
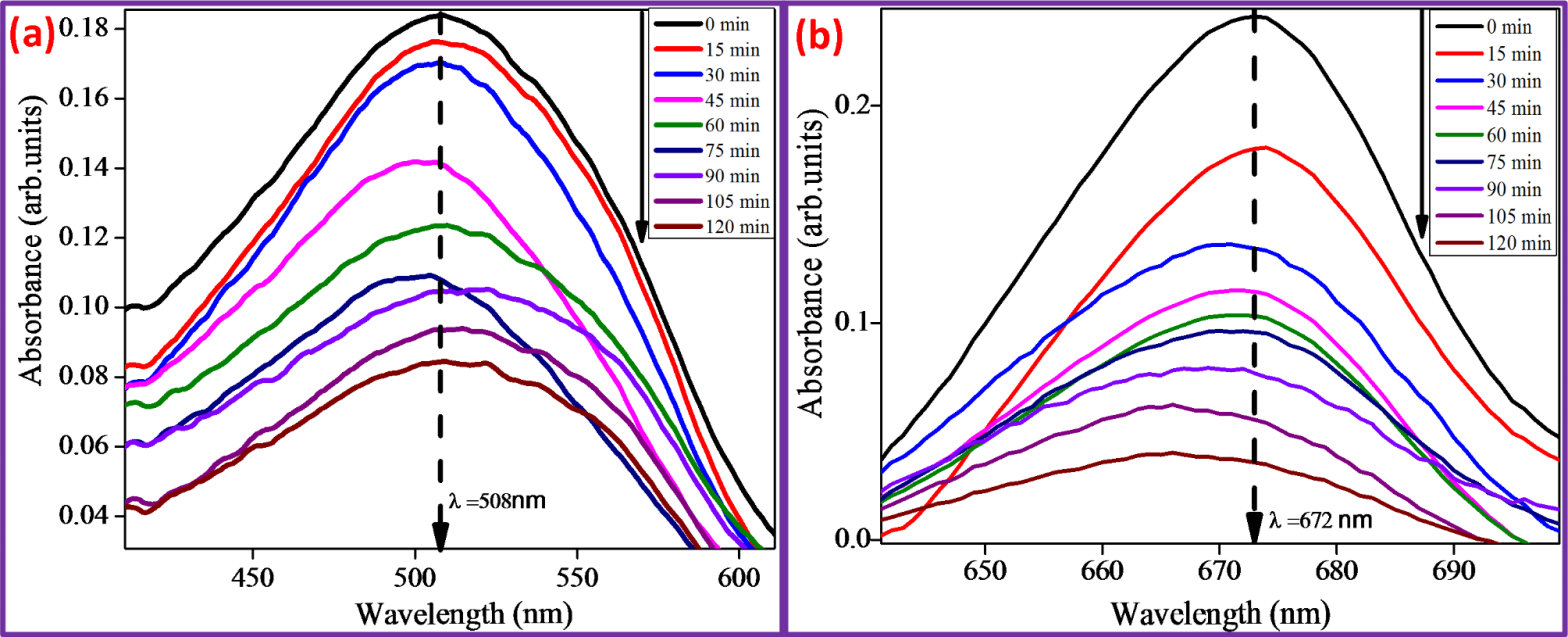
**(a and b)** Absorbance spectra of CR & MB dye for CuO + 5%ZnO under UV light.

The Congo Red (CR) and Methylene Blue (MB) in an aqueous solution were broken down by the photocatalytic experiment of the CuO + 5%ZnO photocatalysts under UV-visible light irradiation. Photocatalytic experiments were performed in a circular glass reactor using a 125 W medium pressure mercury vapor lamp as the source of UV light (280–420 nm) at room temperature (370 nm). In each experiment, 100 mL of CR and MB solutions were combined with 0.1 g of the photocatalyst at a concentration of 15 mg L^− 1^. To prevent heat from the light source, there was a 23 cm gap between the light source and the sample. The reaction mixture was exposed to UV light in open air for 120 min, and 5 mL of the sample solution was removed and measured every 15 min in the 200–800 nm UV-visible range.

**Fig. 6:**
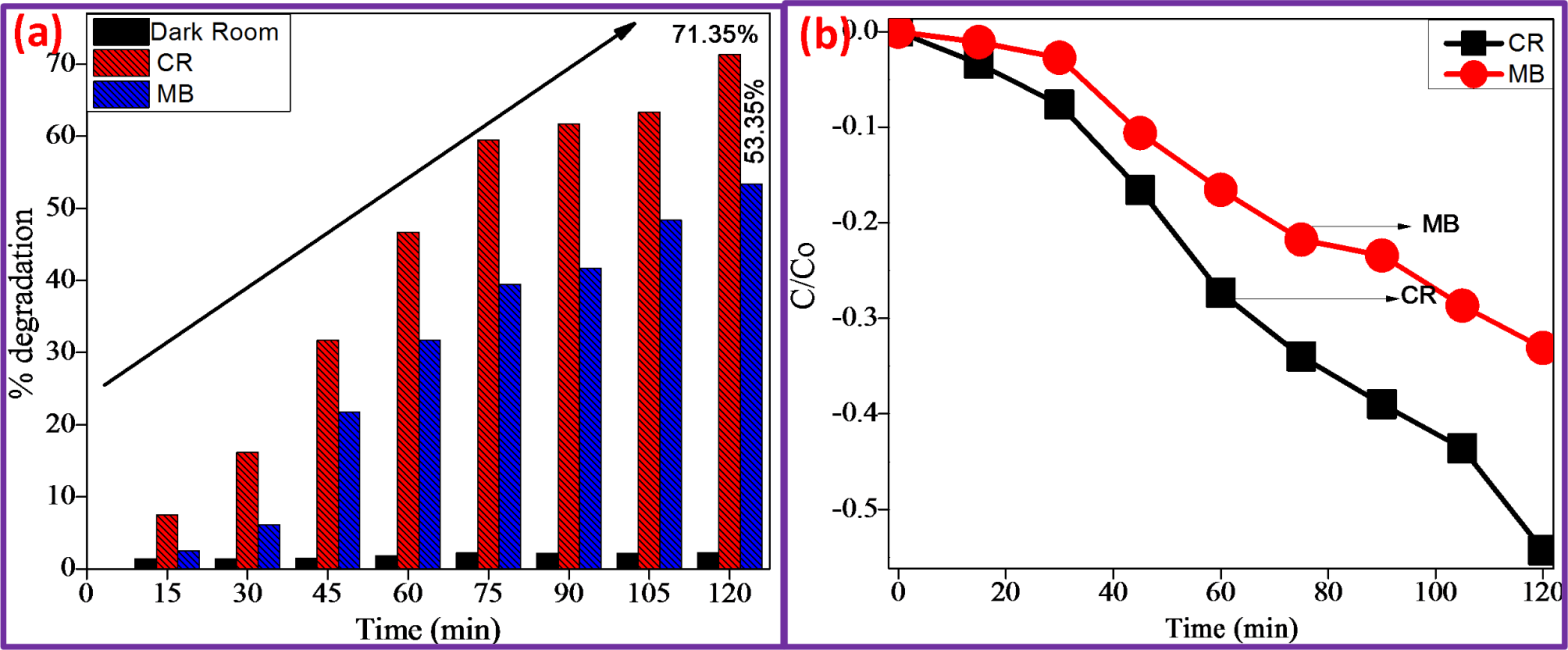
(**a**) % decolourization and (**b**) Plot of C/C0 Vs irradiation time of CR & MB dyes under UV-light CuO + 5%ZnO.

Figure 5a and b show dye decomposition absorbance spectra with maximum wavelengths of 508 and 672 nm for CuO + 5%ZnO. The photocatalytic activity of CuO + 5%ZnO NPs was evaluated by the degradation of CR and MB aqueous solutions. As shown in Fig. 6a, after 120 min of UV irradiation, photodegradation rates of CR and MB dyes decolourized up to 71.35% and 53.35%^39,40^. The percentage degradation of the dyes was determined using Eq. (5).

$$\:\text{\%}\:\text{d}\text{e}\text{g}\text{r}\text{a}\text{d}\text{a}\text{t}\text{i}\text{o}\text{n}=\:\frac{{\text{C}}_{\text{o}}-{\text{C}}_{\text{e}}}{{\text{C}}_{\text{e}}}\times\:100$$

where C_o_-initial dye concentration and C_e_-dye concentration after adsorption at time t (s). Furthermore, the C/Co values were calculated using Eq. (6).

$$\:lo\text{g}\frac{\text{C}}{{\text{C}}_{\text{o}}}=\:-\text{K}\text{t}$$

where Co and C are the concentrations of the dye at time t = 0 min at the time of testing, and k is the first-order rate constant. Figure 6(b) shows that log C/Co and k have a linear relationship, supporting first-order kinetics. For CuO + 5%ZnO, the slope k was estimated for CR and MB under UV light and was 0.004060 m^− 1^, 0.004063 m^− 1^.

**Fig. 7:**
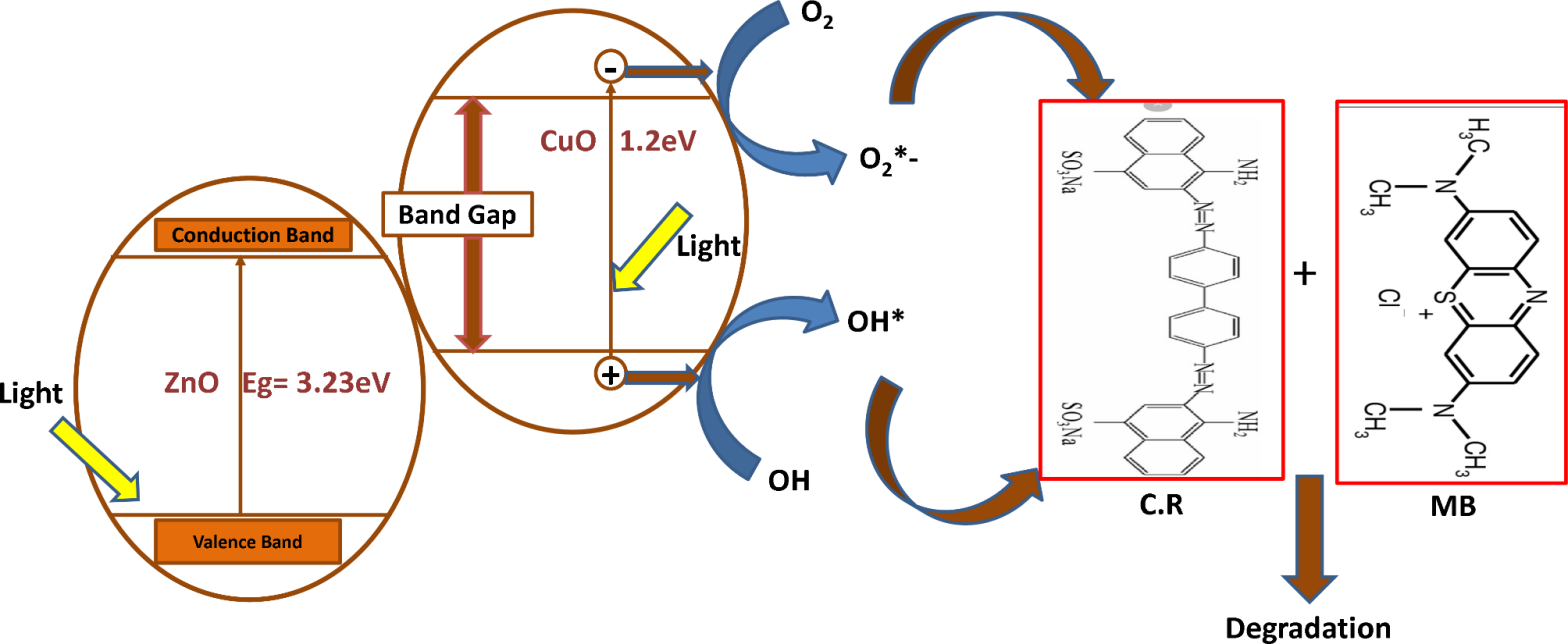
Mechanism for the photocatalytic degradation of CR & MB dye under UV light for CuO+%5ZnO nanocomposite.

As soon as UV falls on CuO + 5%ZnO, a UV photon is absorbed to activate the CuO + 5%ZnO photocatalyst, which leads to the promotion of an electron from the VB to the CB (e^-^CB) and the resulting formation of a gap in the VB (h^+^VB). Finally, the photo-promoter electron or the holes can react with an oxidizable adsorbed species or with a reducible adsorption substrate (often O_2_ in an aerated environment). It consists of four processes that occur after light absorption. The recombination of e–h + pairs occurs primarily at the surface or within the photocatalyst. The charge transferred to the adsorbed species on the surface of CuO has a suitable lifespan for the e-–h + pair as a result of optical light e^-^–h^+^ pair recombination in nanoseconds without such acceptors and donors^41^. A series of reactions occurred in the presence of H_2_O and O_2_. With the help of positive holes, H_2_O is oxidized, and O_2_ is reduced with the help of CB photoelectrons. Reactive O_2_ species were produced, including H_2_O_2_, O_2_.^-^and OH ^•^. and holes may be actively involved in the complete decolorization of the dye solution, as shown in Fig. 7^42^.

**Fig. 8:**
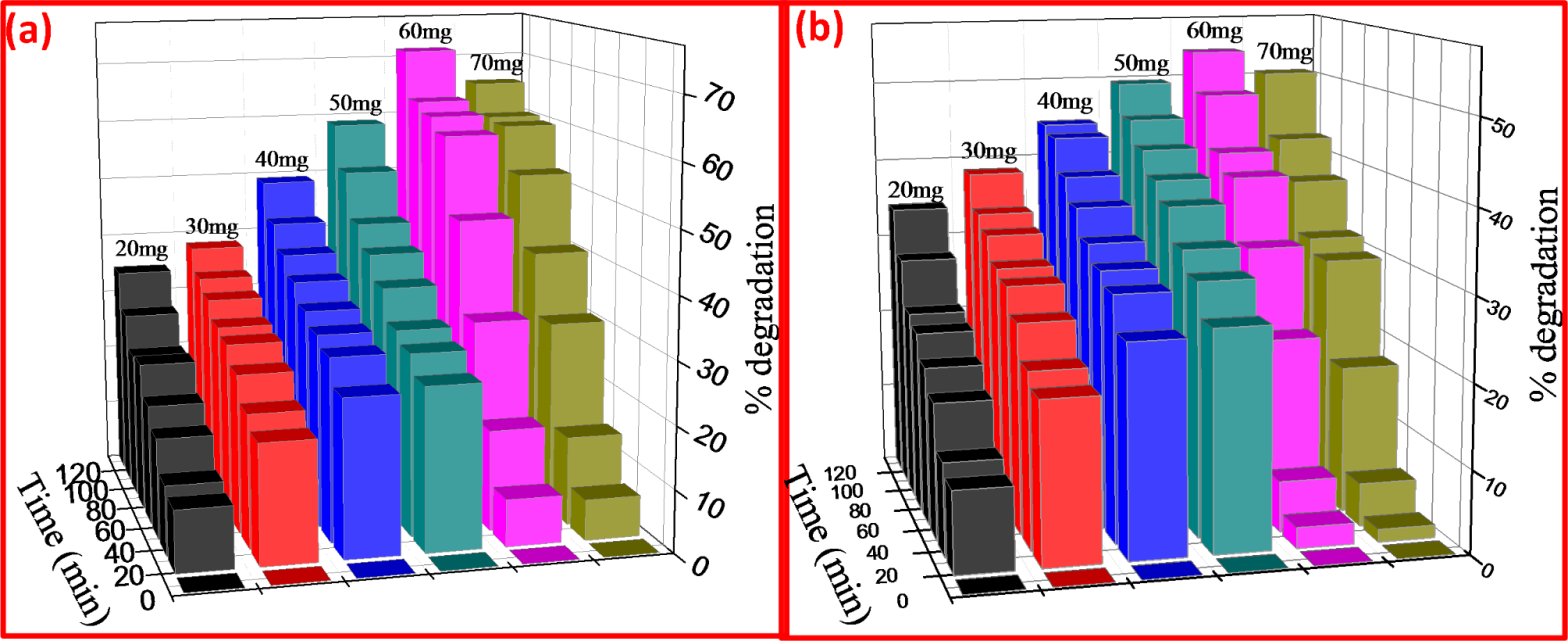
**a and b** Different dye concentration of CR & MB under UV light for CuO + 5%ZnO nanocomposite.

As a consequence, the amount of CuO + 5% ZnO photocatalyst used determines the photodecolorization activity of dye. Under UV light, the decolorization activity of CR and MB dyes was investigated by varying the catalyst dose from 20 to 60 mg and maintaining the concentration (20 ppm) (Fig. 8a and b). Over a 120-min period, a specific dose of catalyst (60 mg) improved the rate of dye photodegradation. A further increase in the catalyst quantity to 60 mg reduced the activity due to the effects of screening and light scattering^43^.

**Fig. 9:**
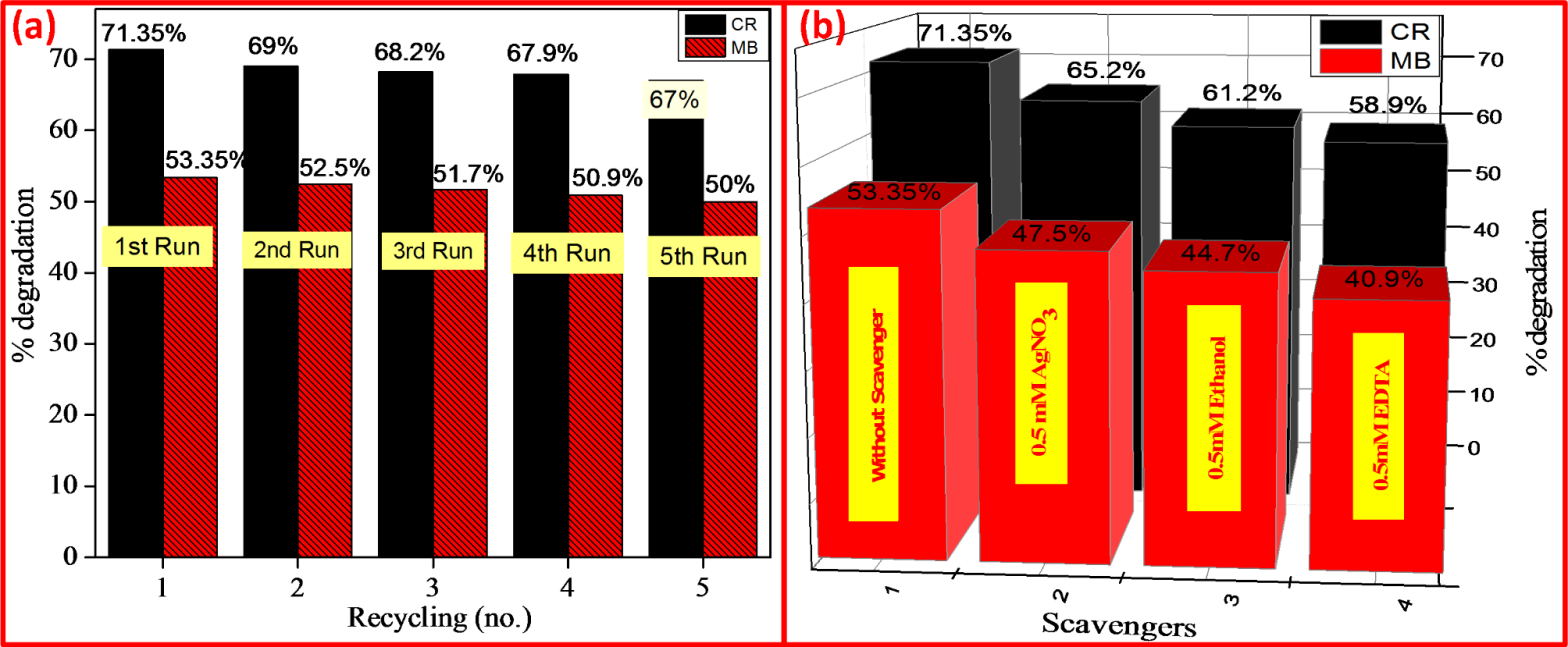
(**a**) Recyclability and (**b**) Scavenging examinations of CR & MB under UV light for CuO + 5%ZnO nanocomposite.

The recyclability of the prepared catalyst is a significant consideration when determining its practical application and scalability. The photocatalysts were separated by centrifugation without washing after each run for the recyclability process, and the degraded CR and MB supernatant was removed before adding fresh CR and MB solution. Five cycle of recycling experiments were carried out to determine the CuO + 5%ZnO sample’s durability and recyclability for photocatalytic degradation Fig. 9(a). CuO + 5%ZnO catalyst could demonstrate some degree of visible-light-induced photocatalytic activity in five subsequent cycling experiments without regeneration. The concentrations of CR and MB decreased in each cycle. Despite this, some photocatalytic activity was lost after recycling, and for the second through five runs, the activity in the first run was higher than that seen in these quential uses at the end of a 2-hour visible light irradiation. The catalyst was still maintained some activity, as seen by the final run’s observation of a 10% degradation of CR and MB, even though it was speculated that the intermediates from the decomposed FB may have blocked the active sites^44,45^.

Scavenging studies were performed to determine efficiency with which highly charged free radicals can remove color from CR and MB dyes when combined with CuO + 5%ZnO photocatalysts and subjected to UV radiation. In Fig. 9(b), we observe that CuO + 5%ZnO is employed in a photo decolouration research of CR and MB under UV light with three different scavengers: AgNO_3_, ethanol, and ethylene-diamine tetra acetic acid (EDTA) (47.5%, 44.7%, and 40.9%, and 65.2%, 61.2%, and 58.9%, respectively. This was performed only for demonstration purposes. All of These results indicate the superior photocatalytic behavior of CuO + 5%ZnO as a catalyst for CR and MB degradation when exposed to UV light. Hence, CuO + 5%ZnO NPs were effective for waste water treatment. AgNO_3_, Ethanol, and benzoic acid were added, and these substances scavenged electrons, holes, hydroxyl radicals (^•^OH), and superoxide radicals (O_2_^−•^). As a result, CR and MB are degraded more quickly than holes, electrons, and superoxide radicals.

###  Electrochemical performance of ZnO doped CuO

**Fig. 10:**
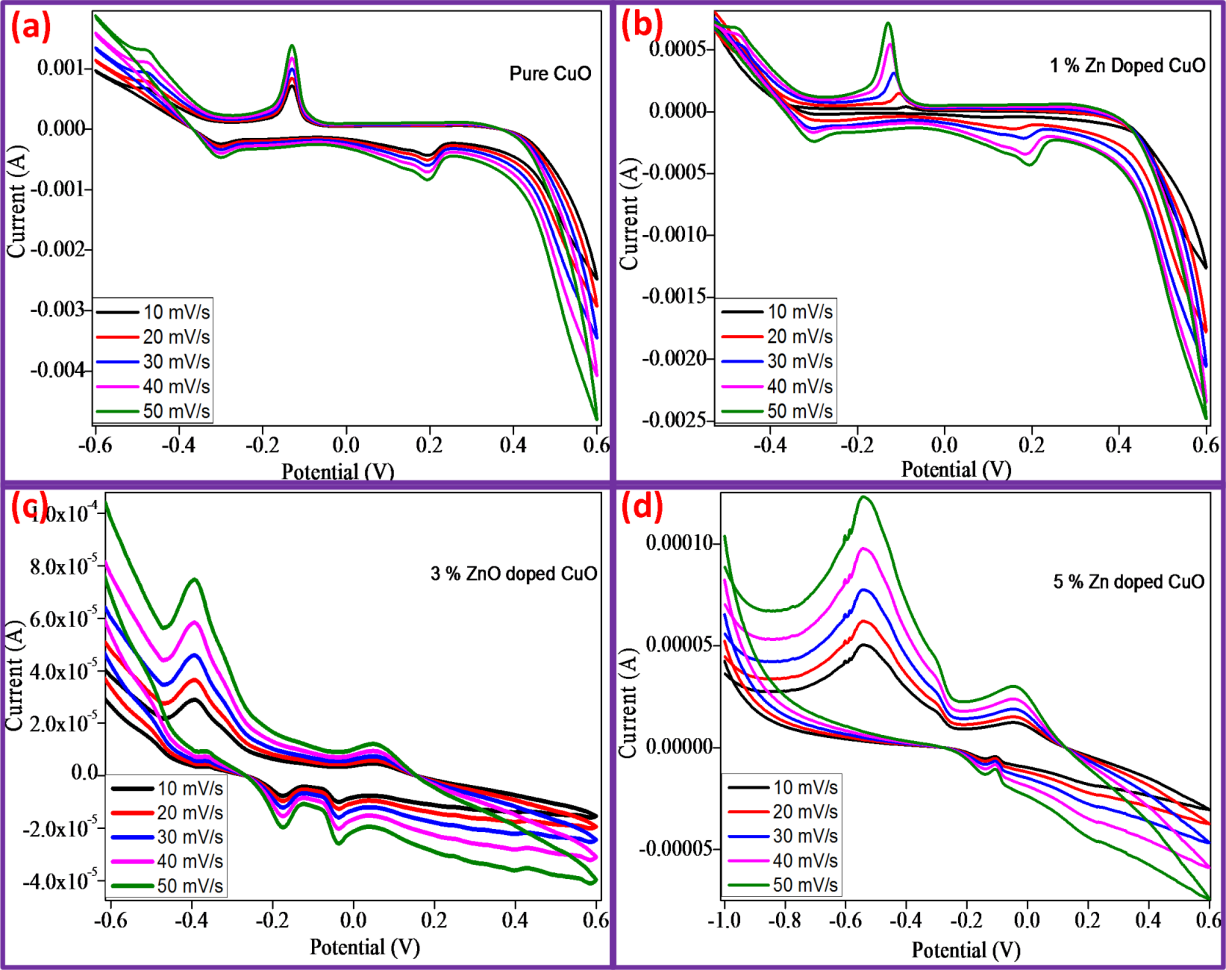
Cyclic voltammogram of (**a**) CuO, (**b**) CuO + 1%ZnO, (**c**) CuO + 3%ZnO and (**d**) CuO + 5%ZnO samples at various scan rates vs. Ag/AgCl electrode.

Cyclic voltammetry (CV) to determine the capacitive performance and electrochemical properties of super capacitor electrodes is a helpful approach for CuO, CuO + 1%ZnO, CuO + 3%ZnO, and CuO + 5%ZnO, as shown in Fig. 10a–d. Three combinations of electrode systems allow for the performance of CV analysis (-0.6 V to + 1.0 V (vs. Ag/AgCl)). Figure 10a–d depict the behavior of CuO and CuO-ZnO composites in 1 M KOH as an electrolyte at various scan speeds (10, 20, 30, 40, and 50 mV/s)^46^. The electron transfer between the redox species at the working and counter electrodes generates a current that is carried through the solution by the diffusion of ions. Good electrode stability was demonstrated by the anodic and cathodic peak positions of the electrode, which did not change significantly with the growth cycles^47^.

According to the Randles-Sevcik equation for a reversible process, the current height is represented by Eqs. (7 and 8)

7 $$\text{i}\text{p}=2.69\:\times\:{10}^{5\:}\times\:{n}^{3/2}\:\times\:A\:\times\:{D}^{1/2}\times\:{C}_{0}\:\times\:{v}^{1/2}$$

where n is the number of electrons, A is the extent of the electrode, is the diffusion co-efficient, ν-scanning rate and C_0−_initial concentration.

8 $$\:{\text{C}}_{0}=\frac{\rho\:}{M}$$

Where ρ- theoretical density and M- molar mass of samples.

**Fig. 11:**
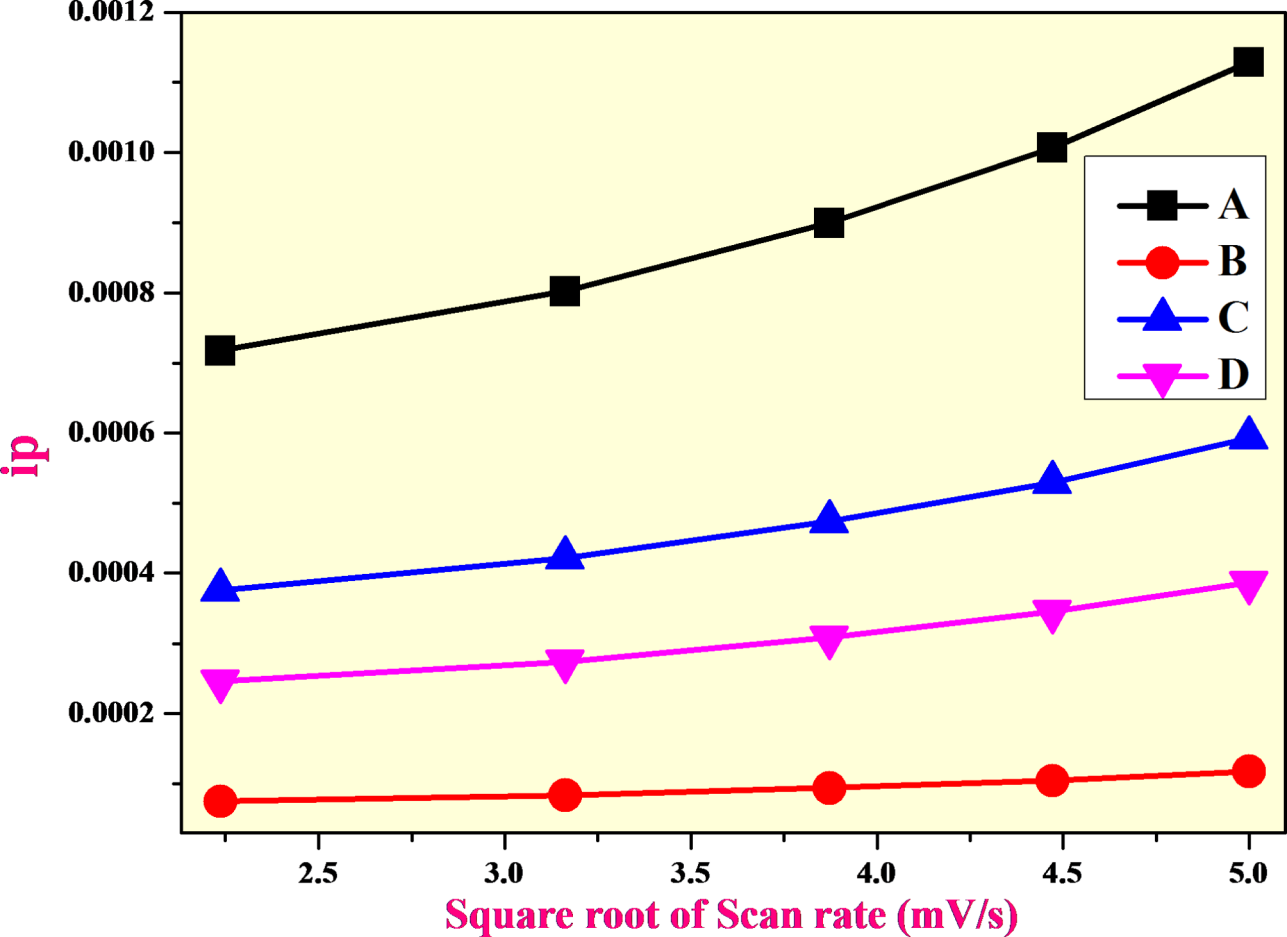
Relationship between the anodic peak current (ip) and the square root of the scan rate (υ½) for (**a**) CuO, (**b**) CuO + 1%ZnO, (**c**) CuO + 3%ZnO and (**d**) CuO + 5%ZnO.

The relationship between the cathodic peak current (ip) and square root of the electrode sample scan rate (v1/2) is shown in Fig. 11. CuO and CuO-ZnO electrode reactions are constrained by hydrogen diffusion, as shown by the strong linear relationship between ip and 1/2. Using Eq. (7) and slope of fitted line in Fig. 11, proton diffusion coefficient (D) for CuO, CuO + 1%ZnO, CuO + 3%ZnO, and CuO + 5%ZnO electrode materials calculated to be 2.470 × 10^− 6^ cm^2^s^− 1^, 2.528 × 10^− 5^ cm^2^s^− 1^, 1.759 × 10^− 4^ cm^2^s^− 1^ and cm^2^s^− 1^. Out Of these, the CuO + 5%ZnO is comparatively greater than that of other electrode materials^48,49^. Table 2 shows the comparison of electrochemical performances of electrodes.

**Table 2:** Comparisons of electrochemical performances of electrodes.

Name of the electrode	Diffusion coefficient (D) cm^2^s^− 1^	Other literature Diffusion coefficient (D) cm^2^s^− 1^
CuO	2.470 × 10^− 6^	3.804 × 10^− 6^
CuO + 5%ZnO	2.528 × 10^− 5^	2.424 × 10^− 4^
CuO + 10%ZnO	1.759 × 10^− 4^	7.685 × 10^− 3^
CuO + 15%ZnO	6.048 × 10^− 5^	4.534 × 10^− 4^

**Fig. 12:**
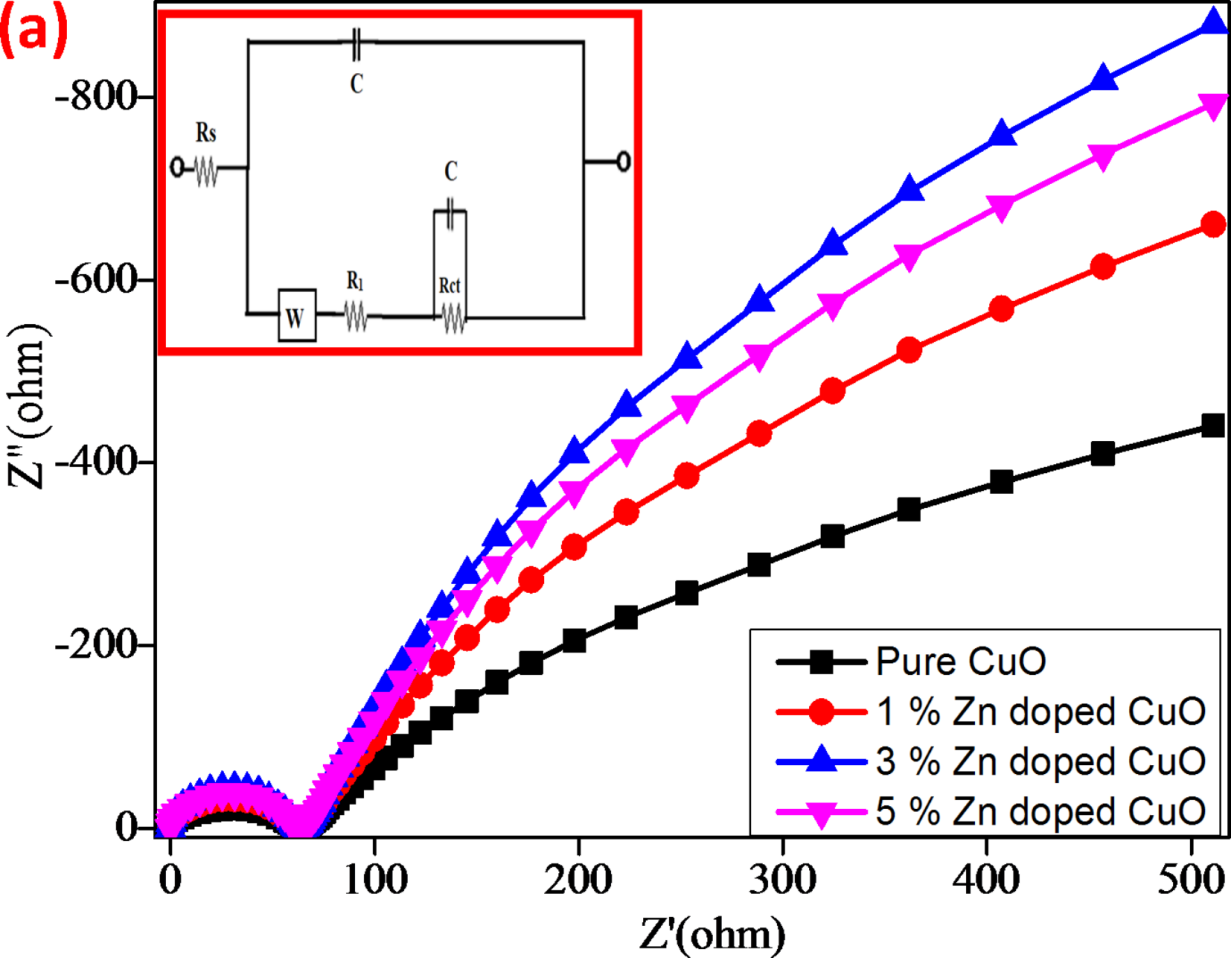
Nyquist plot with simulation of (**a**) CuO, (**b**) CuO + 1%ZnO, (**c**) CuO + 3%ZnO and (**d**) CuO + 5%ZnO electrodes.

In Fig. 12, the impedance spectra of the charge-transfer resistance and bulk resistance demonstrate the properties of the prepared electrodes. The creation of a double layer at the electrode-electrolyte interface was confirmed by a nearly vertical line followed by a line with an approximate slope of unity in the lower frequency range^50^. We discovered that different parameters, such as the charge transfer resistance (Rct) and electrode capacitance (Cdl), are tabulated alongside the total capacitance values using the intercepts on the real axis (Table 2). The high capacitance and low resistance of the electrodes made of for CuO, CuO + 1%ZnO, CuO + 3%ZnO, and CuO + 5%ZnO may be the result of the electrodes’ nanocomposite pores being well matched to the ions in the electrolyte (Table 3).

**Table 3:** EIS data’s for CuO, CuO + 1%ZnO, CuO + 3%ZnO and CuO + 5%ZnO electrodes.

Name of the electrode	Charge-transfer resistance (*R*_Ct_) (Ω)	Capacitance of double layer (C_dl_) (F)
CuO	135.76	3.804 × 10^− 6^
CuO + 5%ZnO	32.28	2.424 × 10^− 4^
CuO + 10%ZnO	33.61	7.685 × 10^− 3^
CuO + 15%ZnO	62.82	4.534 × 10^− 4^

The impedance spectra in Fig. 12 were additionally analyzed using a fitting technique with the help of the modified Randal equivalent circuit, which includes Rs (solution resistance), Cdl (double layer capacitance), Rct (charge-transfer resistance), R1 is the leakage resistance, and W is the Warburg component, as shown in the inset of Fig. 12^51^. Rs indicates the resistance of the electrode and current collector.

The charge transfer resistance (Rct) and double layer capacitance (C) values were measured using a two-dimensional figure at high frequencies, as observed in the resistance plot. From these plots, it is clear that the charge-transfer resistance is low in electrode C, followed by an increase in the capacitance of the electrode. From this knowledge, we clarified that the electrochemical behavior of the electrode C (CuO + 3%ZnO) is superior to that of other electrodes^52,53^.

###  Sensor studies

**Fig. 13:**
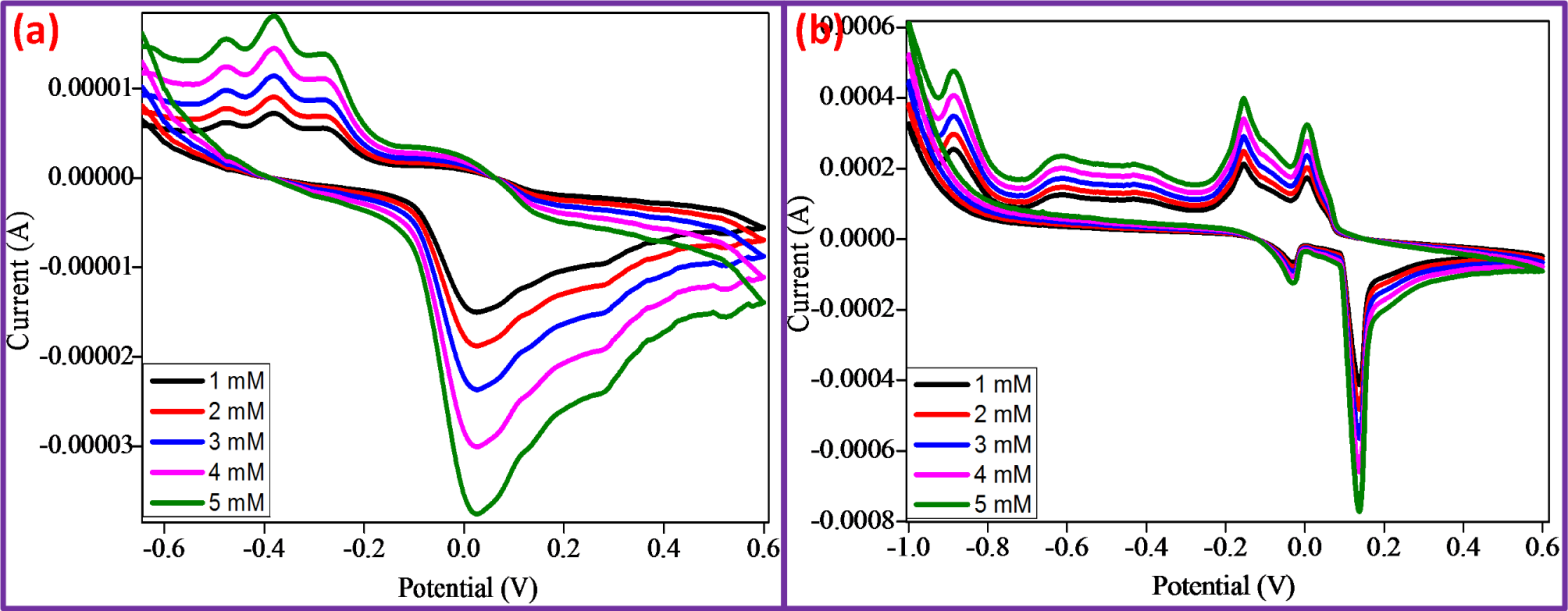
**a and b** Cyclic voltammograms of CuO + 5%ZnO NPs showing the detection of metals, concentration range 1–5 mM, (**a**) Glucose, (**b**) Ascorbic acid.

In addition, we have also extended the sensing investigation of the CuO + 5%ZnO NPs for detection of metal ions like glucose and ascorbic acid in alkaline media at concentrations ranging from 1 to 5 mM. For sensing (Fig. 13a), the anodic and cathodic peak position changed to 0.02 V and − 0.4 V respectively, while increase in cathodic current was observed with increasing glucose content. Similarly, in ascorbic sensing (Fig. 13b), minor peaks appeared on both sides: an oxidation peak at 0.18 V and a reduction peak at -0.21 V. From Fig. 13a and b, it is confirmed that the prepared CuO + 5%ZnO NPs electrode appeared to be the most effective material for sensing Glucose & Ascorbic acid.

In addition, we noticed the appearance of sharp oxidation peak at 0.18 V and reduction peak at -0.21 V in ascorbic acid^53,54^ (Table 4).

**Table 4:** Comparison table for degradation and sensing experiment.

Name of the electrode	Degradation dye CR and MB	Previous Degradation dye *p*-Nitrophenol	Sensor for Glucose & Ascorbic acid
CuO + 5%ZnO	71.35 & 53.35%	98.32%	Oxidation peak at 0.18 V and reduction peak at -0.21 V

## Conclusions

ZnO-doped CuO nanocomposite (CuO-ZnO NPs) of 1, 3, and 5 mol% by the solution combustion method using ODH as a fuel (oxlyl-hydrazide) at 500 °C and calcining at 1000 °C for two hours. The structural, photocatalytic, and electrochemical properties were examined using experimental and theoretical methods. For pure CuO and CuO-ZnO NPs of 1, 3, and 5 mol%, the XRD (X-ray diffraction) patterns showed a crystallite size (D) range of 23 nm. The optical energy bandgap (Eg) of the NPs was estimated to be between 2.1 and 2.5 eV. The photocatalytic degradation of CuO + 5%ZnO NPs on CR & MB dye was evaluated under UV light irradiation. The CuO + 5%ZnO NPs composite degraded 71.35 and 53.35% of the dye when exposed to UV radiation. Furthermore, the CV measurements of CuO + 5%ZnO NPs were extended for heavy metal detection, such as glucose and ascorbic acid, by chemical sensor studies. A drastic variation in oxidation and reduction peak positions was observed, confirming that the prepared carbon paste electrodes using CuO + 5%ZnO NPs are effective materials for sensor applications. Thus, the reported results confirm that the prepared CuO + 5%ZnO NPs can be used as a promising substance for electrochemical, sensor, heavy metal detection in industrial waste water treatments for environmental remediation by using several chemicals and sensors.

## Data Availability

The datasets generated or analyzed during the current study are available from the corresponding author upon reasonable request.
